# The value of magnetic resonance imaging of the optic nerve for the diagnosis of multiple sclerosis in patients with optic neuritis

**DOI:** 10.1007/s00415-024-12801-7

**Published:** 2025-01-15

**Authors:** Gorm Pihl-Jensen, Jette Lautrup Frederiksen

**Affiliations:** Department of Neurology, Clinic of Optic Neuritis and Danish Multiple Sclerosis Center, Rigshospitalet-Glostrup, Valdemar Hansens Vej 13, 2600 Glostrup, Denmark

**Keywords:** Multiple sclerosis, MRI, Optic nerve lesions

## Abstract

**Background:**

Although optic neuritis (ON) is common in multiple sclerosis (MS), lesions of the optic nerve are not included as an anatomical substrate for dissemination in space and time (DIS and DIT).

**Objective:**

To assess the increase in sensitivity of including MRI lesions of the optic nerve for the diagnosis of MS in patients with ON.

**Methods:**

We included patients consecutively referred with first time, monosymptomatic ON, with no known cause of the ON, who underwent orbital MRI including fat suppressed T2 and T1-sequences with and without gadolinium contrast.

**Results:**

One hundred and twenty patients were included. Optic nerve T2 lesions and/or T1-contrast enhancement was shown in 104 patients. Sixty-three patients were diagnosed with MS at baseline. Nine patients developed MS during follow-up. The inclusion of optic nerve MRI lesions led to the diagnosis of 8 additional patients and increased sensitivity to 0.99 (95% CI 0.96–1.00) compared to 0.88 (95% CI 0.79–0.95) for 2017 criteria, while decreasing the specificity to 0.81 (95% CI 0.70–0.92) compared to 1.00.

**Conclusion:**

Amending the diagnostic criteria for MS to include MRI lesions of the optic nerve as a substrate for DIS and DIT may increase sensitivity and lead to more rapid diagnosis of MS.

## Introduction

Multiple sclerosis (MS) is a chronic inflammatory disease affecting various parts of the central nervous system [1]. MS is characterized by disease activity disseminated in space (DIS) and disseminated in time (DIT) [2]. The current diagnostic criteria of relapsing MS, the 2017 revisions of the McDonald criteria (2017_McD_), reflect this as DIT and DIS may be achieved clinically or due to MRI findings [2]. In the case of a typical clinical isolated syndrome (CIS), i.e. a clinical episode similar to a typical MS relapse and no known MS or prior relapses, an MS diagnosis can be achieved on the basis of demonstration of DIS and DIT due to radiological evidence [2], mainly MRI lesion detection 2 out of 4 anatomical sites typical of MS (periventricular, cortical or juxtacortical, infratentorial or in the spinal cord) [2].

Affliction of the optic nerve and anterior visual pathway is common in MS [3]. About 20% of MS patients report optic neuritis (ON) as the onset symptom [4] and up to 70% of MS patients will experience clinical ON [3]. Over half of ON patients will receive an MS diagnosis at presentation or later [5]. ON represents an appealing model for the MS relapse due to its strict topographical arrangement and a wealth of sensitive tests to demonstrate anterior visual pathway damage [6]. Orbital MRI sequences (including fat-suppressed, T1 coronal plane, post contrast and T2 sequences) may demonstrate acute ON in more than 80–90% of cases [7, 8]. ON may in this case be presented with T2-hyperintensive lesions and contrast-enhancing T1 lesions of the optic nerve, optic disc or optic chiasm [8], but orbital specific MRI sequences are not routinely used at all clinics investigating demyelinating disorders.

In spite of the intricate relationship between ON and MS, lesions of the optic nerve were not included as an anatomical substrate due to a perceived lack of evidence [2]. Proposals have been presented made to include the optic nerve as a fifth site of DIS by demonstrating affliction of the optic nerve by MRI [9–11], optical coherence tomography (OCT) [12] and visual evoked potentials (VEP) [11, 13]. However, further evidence is required to validate the inclusion of the optic nerve and anterior visual system in the diagnostic criteria for MS.

With this study we therefore aimed to examine the diagnostic value of orbital MRI in ON and to examine whether including MRI lesions of the optic nerve increases sensitivity for the diagnosis of MS in patients with ON.

## Methods

### Subjects

The study was performed in a prospective cohort of patients referred to the Clinic of Optic Neuritis, Department of Neurology, Rigshospitalet-Glostrup Denmark, referred with first time ON and no known diagnosis of MS or CIS prior to referral. The clinical diagnosis of ON was made by a professor of Neurology (co-author Jette Frederiksen) and was based on symptoms and signs e.g., blurry vision, affliction of contrast vision, color vision and visual fields, periorbital pain accentuated by ocular movements, and in some cases presence of swollen discs [14]. Patients eligible for inclusion in this study had undergone MRI with orbital sequences. Patients aged 18 years or above were eligible for inclusion in the study. Through a rigorous, standardized diagnostic evaluation, we excluded patients with other identifiable causes of ON. This included a wide array of laboratory analyses including for myelin oligodendrocyte glycoprotein and aquaporin-4 antibodies. In patients who did not receive a MS diagnosis at baseline evaluation, the subsequent relapse or MRI activity was recorded. End of follow-up time was set at 1st of March 2024.

### Test methods

MRI was performed on a 3 T Phillips Achieva MRI scanner using a standardized clinical protocol employing axial and coronal T1-weighted sequence with fat suppression before and after gadolinium contrast injection as well as coronal T2 weighted sequence with fat suppression. Cerebral MRI was performed using axial and transversal T2, FLAIR and DWI as well as T1-weighted scan pre- and post-gadolinum contrast injection. MRI of the spinal cord was performed using axial and transversal T2 and FLAIR sequences. T1 contrast enhancement was not routinely employed in spinal MRI.

### Statistical analysis

We examined the diagnostic value of orbital MRI in ON and evaluated the performance of the 2017 McDonald criteria (2017_McD_ [2]) and a revised version of these criteria, 2017_ON+,_ where we included the optic nerve as a fifth anatomical substrate for DIS and where gadolinium enhancing lesions (GEL) of the optic nerve were to be included in assessment of DIT (i.e. simultaneous presence of contrast enhancing and non-contrast enhancing lesions [2]). Comparison between the performance of the 2017_McD_ and 2017_McDON+_ criteria was performed with the McNemar test of binary tests with paired data. For the two sets of criteria, we estimated sensitivity, specificity, positive predictive value, and negative predictive value with estimated 95% confidence intervals. In cases where 2017_McD_ had not yielded an MS diagnosis at baseline, the defining outcome of a certain MS diagnosis was defined by fulfilling MS criteria during the follow-up time period, either due to new T2 lesions (demonstrating DIS and DIT) or a second relapse.

Quantitative data are presented as mean (SD) or median (IQR) according to distribution of data.

Statistical analysis was performed in R (v.3.5.1).

### Ethical considerations

The study has been approved by the Regional Scientific Ethical Committee, Capital Region, Denmark (protocol H-4-2014-095) and adhered to the tenets of the 1964 Helsinki declaration and later amendments. All participants provided written consent prior to inclusion.

## Results

**Fig. 1:**
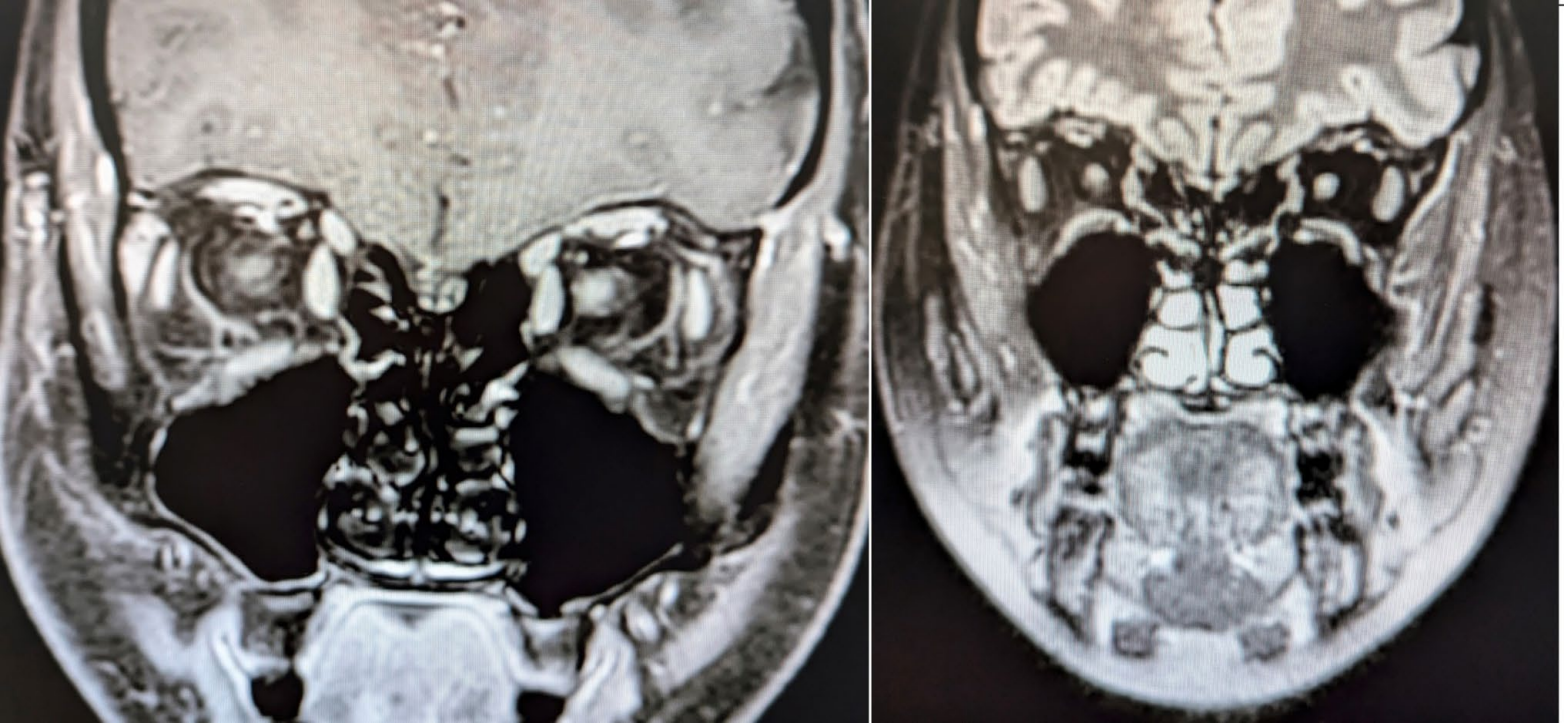
Optic nerve lesion shown on orbital MRI (coronal T1-weighted sequence after gadolinium contrast injection and coronal T2 weighted sequence)

One-hundred and twenty patients were included in the study out of which 74 were women (Table 1). All 120 patients underwent orbital MRI sequences upon referral, median time from onset to MRI was 19 days (IQR:17). In 103 of the 120 patients hyperintensive T2-lesions were shown in the afflicted optic nerve corresponding to 86%, whereas GEL of the optic nerve (Fig. 1) was demonstrated in 86 of 115 patients corresponding to 75%. In five patients gadolinium contrast was not infused either per patient request or due to previous allergic reactions. In total, T2 hyperintensive lesions and/or GEL were demonstrated in 104 of 120 patients. T2 hyperintensive lesions and GEL were shown in only 5 and 4 contralateral eyes, respectively.

**Table 1:** Characteristics of the included patients

Characteristics of the included patients	
*n*	120
Age in years [mean(SD)]	33.7 (9.3)
Female sex [*n* (%)]	74 (70)
Time between ON onset and MRI in days [median (IQR)]	19 (17)
Follow-up time in days [median (IQR)]	158 (876)
CSF oligoclonal bands present [*n* (%)]^a^	89 (77.4)
MRI baseline showing ≥ 1 T2 WML brain [*n* (%)]	86 (71.7)
MRI baseline showing ≥ 1 T2 WML spinal cord [*n* (%)]^b^	44 (41.5)
MRI baseline DIS criteria fulfilled [*n* (%)]	
0 of 4	41 (34.2)
≥ 1 of 4	79 (65.8)
≥ 2 of 4	60 (50.0)
≥ 3 of 4	33 (27.5)
4 of 4	18 (15.0)
MRI T2 hyperintensive lesion optic nerve ON eye [*n* (%)]	103 (85.8)
MRI GEL optic nerve ON eye [*n* (%)]	86 (74.8)

*ON* Optic neuritis, *CSF* Cerespospinal fluid, *WML* White matter lesion(s), *GEL* Gadolinum enhancing lesion(s), *DIS* Dissemination in space

^a^5 patients did not agree to a spinal tap

^b^MRI of the spinal cord was not performed in 14 patients

**Fig. 2:**
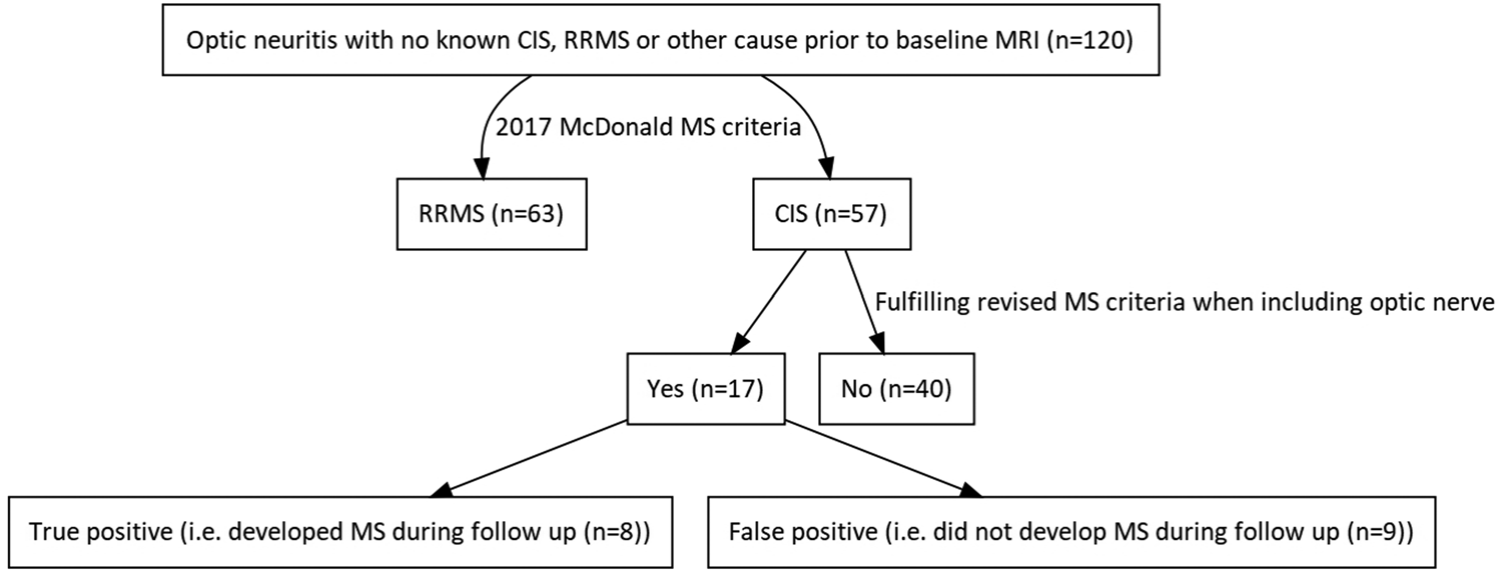
Flowchart displaying the proportion of optic neuritis patients fulfilling 2017 McDonald criteria for multiple sclerosis at baseline and proportion of patients not fulfilling the 2017 criteria, who fulfilled criteria when including optic nerve lesions. CIS: clinically isolated syndrome. RRMS: relapsing remitting multiple sclerosis

In total 72 patients were classified as RRMS during the study out of which 63 patients were diagnosed at baseline and 9 patients during follow according to the 2017_McD_-criteria (Fig. 2). In patients who were not diagnosed with MS at baseline, nine of 57 patients were treated with disease modifying therapies during follow-up including six of the nine patients with an eventual MS diagnosis. The 2017_ON+_-criteria identified eight of the nine patients at baseline. Two of eight patients correctly identified by the revised criteria received a definite MS diagnosis due to a new relapse during follow-up, while the remaining six of eight patients were diagnosed with MS due to a new MRI fulfilling criteria for DIS and DIT due to new T2 lesions and/or GEL. Of the eight patients who would have been diagnosed by the 2017_ON+_ criteria only 2 fulfilled DIS criteria of the 2017_McD_ criteria. In six of eight patients oligoclonal bands were demonstrated (Table 2), in all of these patients, GEL was shown in the symptomatic optic nerve.

**Table 2:** Characteristics of the patients who were diagnosed with MS at baseline according to the 2017 McDonald criteria (2017_McD_) and patients who would have been diagnosed with MS at baseline with the revised criteria (2017_ON+_). Significant differences between the groups are indicated in bold

	2017_McD_ criteria true positive at baseline	2017_McD_ criteria false negative and 2017_ON+_ criteria true positive at baseline	*p* value for difference
*n*	63	8	
Age [mean (SD)]	34.0 (9.7)	34.4 (8.3)	0.74
Female sex [*n* (%)]	46 (73.0)	7 (87.5)	0.47
Days_from_onset [mean (SD)]	29.17 (37.7)	42.25 (52.2)	0.60
CSF oligoclonal_bands present [*n* (%)]	62 (100.0)	6 (75.0)	** < 0.001**
MRI baseline showing ≥ 1 T2 WML spinal cord [*n* (%)]	39 (68.4)	4 (50.0)	** < 0.001**
MRI baseline total T2 WML brain [median(IQR)]	6 (18)	2 (5)	**0.040**
MRI T2 hyperintensive lesion optic nerve ON eye [*n* (%)]	60 (95.2)	8 (100.0)	0.54
MRI GEL optic nerve ON eye [*n* (%)]	50 (83.3)	8 (100.0)	0.22

*CSF* Cerebrospinal fluid, *WML* Whiter matter lesion(s), *GEL* Gadolinum enhancing lesion(s)

The eight patients who would have been diagnosed with the 2017_ON+_-criteria, were in fact diagnosed with definite MS at a median duration of 293.5 days (IQR: 102.3) from baseline. Characteristics of patients diagnosed at baseline by the 2017_McD_ criteria and patients who would additionally been diagnosed with the 2017_ON+_ criteria are outlined in Table 2. Notably, patients diagnosed at baseline by the 2017_McD_ included a significantly higher proportion of patients with oligoclonal bands and ≥ 1 T2 hyperintensive lesions on MRI and a significantly higher median number of T2 hyperintensive lesions.

Sensitivity for a diagnosis of MS during follow-up was significantly higher with the 2017_ON+_ (0.99 (95% CI 0.96–1.00) compared to the 2017_McD_ criteria 0.88 (95% CI 0.79–0.95), *p* value for difference 0.0047 (Table 3).

**Table 3:** Diagnostic accuracy values of the 2017 McDonald criteria for multiple sclerosis (2017_McD_) and the proposed revised 2017 criteria (2017_ON+)_ including MRI lesions of the optic nerve in the demonstration of dissemination in time and space

	2017_McD_ criteria	2017_ON+_ criteria
Sensitivity	0.875 (95% CI 0.799–0.951)	0.986 (95% CI 0.959–1.000)
Specificity	1.000 (95% CI 1.000–1.000)	0.813 (95% CI 0.702–0.923)
Positive predictive value	1.000 (95% CI 1.000–1.000)	0.888 (95% CI 0.818–0.957)
Negative predictive value	0.842 (95% CI 0.747–0.937)	0.975 (95% CI 0.927–1.000)

Meanwhile, specificity was significantly higher with the 2017_McD_ criteria compared with the revised criteria 1.00 vs 0.81 (95% CI 0.70–0.92), *p* value for difference 0.0027. Correspondingly, the 2017_ON+_ criteria demonstrated a higher negative predictive value 0.975 (95% CI 0.93–1.00) compared to the 2017_McD_ criteria 0.84 (95% CI 0.75–0.94) with a lower positive predictive value (Table 3).

## Discussion

We present here a large, single center study of ON patients and demonstrate the diagnostic value of dedicated orbital MRI sequences in these patients. Our findings confirm prior studies [7, 9, 11] and add to the existing evidence of the role of dedicated orbital MRI sequences in the evaluation of patients with ON.

The current diagnostic criteria for relapsing MS focus on clinical and radiological substrates for DIS and DIT, but neglect the anatomical substrate of lesions in the optic nerve and anterior visual system. Therefore, in patients presenting with ON, in contrast to other CIS presentations, a lesion must be detected in two additional anatomical localizations to fulfill the diagnostic criteria [2]. Thus, in this study, we focused on patients referred with a first time ON, no known diagnosis of MS or CIS and no discernible alternative cause of the ON. We aimed to ascertain the increased value of performing orbital sequence MRI in this particular patient group. Traditionally, ON was thought to carry a more favorable prognosis regarding MS risk than other CIS [4], however, adjusting for baseline factors and MRI lesions, MS risk may be at the level of other types of CIS [15, 16]. Taken together with the intricate relationship between MS and ON and the anterior visual system, the absence of optic nerve in the diagnostic criteria of MS seems counterintuitive.

Adding the optic nerve as an anatomical substrate in the MS criteria should emphasize caution in excluding cases with likely differential diagnoses or more likely causes of ON [2], especially in cases where only medullary lesions and affliction of the anterior visual system are shown. Further studies are warranted to study the natural concern of over- and misdiagnosis.

DIT has been a main characteristic in MS disease course and hence MS criteria [2, 17, 18], however, there is an increasing focus on establishing this criterion at the stage of first demyelinating event to accommodate the increasing evidence of early, active treatment of RRMS [18]. Notably, in our cohort, eight patients would have received an MS diagnosis at baseline if the optic nerve were included as an anatomical substrate. Of the eight patients, six showed oligoclonal bands and all six of these patients included GEL of the symptomatic optic nerve demonstrating the continually important role of oligoclonal bands, or corresponding CSF findings, in diagnosing MS. Including GEL of the symptomatic, or asymptomatic, eye may however be useful in cases where lumbar puncture is unwanted, contraindicated or refused by the patient.

Our findings suggest an earlier MS diagnosis is achievable in some patients presenting with a monosymptomatic ON and no prior clinical activity suggestive of MS. An earlier MS diagnosis is beneficial in many cases with regards to treatment decisions, insurance matters and to reduce the time spent in uncertainty for patients and relatives. This is especially true in the case of ON which traditionally has been regarded with a more uncertain MS risk than other types of CIS.

Contrary to previous studies [9–11] we included a homogenously examined cohort exclusively of ON patients to examine the diagnostic value of the amended criteria specifically in this patient group. Our single center cohort included a representative group of patients consecutively referred, from a geographically well-defined area of Eastern Denmark, to a public hospital for evaluation. Our cohort had a shorter time from onset than comparable studies [9–12] and provide real-world evidence that even in the acute setting of ON, consideration of revision of the diagnostic criteria for MS, including the optic nerve, is warranted.

Our study does contain some limitations. We did not include OCT signs of optic nerve affliction since the vast majority of patients were examined within the acute phase of ON where full RNFL and GCL thinning has not occurred [19]. We also did not include VEP measurements in the study since the majority of patients were examined at an early phase of ON where the value of VEP measurements may be limited due to conduction block [20–22]. Both OCT and VEP constitute important methods to demonstrate affliction of the optic nerve and anterior visual pathway. Previous studies have indicated their value in this regard especially in patients with other manifestations of CIS [11–13, 23]. Furthermore, our study had a shorter follow-up time than comparable studies [9–12], however the large proportion of patients receiving a MS diagnosis during follow-up in our cohort, indicates that extending follow-up time would only increase this proportion slightly.

In summary, our study found that in a cohort of first time ON, with no known diagnosis of CIS, MS or other cause of ON, including optic nerve lesions to the McDonald MS criteria, increases sensitivity and negative predictive value and provides a markedly earlier MS diagnosis in some patients while decreasing specificity.
